# CRITICAL OR ENCOURAGING? CORRELATES OF FAMILY CAREGIVERS’ DEMENTIA MANAGEMENT STRATEGIES

**DOI:** 10.1093/geroni/igae098.2410

**Published:** 2024-12-31

**Authors:** Amanda Leggett, Jonathan Troost, Sophia Tsuker, Jennifer Miner, Noelle Carlozzi

**Affiliations:** Wayne State University, Detroit, Michigan, United States; University of Michigan, Ann Arbor, Michigan, United States; Wayne State University, Detroit, Michigan, United States; University of Michigan, Ann Arbor, Michigan, United States; University of Michigan, Ann Arbor, Michigan, United States

## Abstract

Family caregivers approach caregiving challenges with a variety of management strategies which may be adaptive or maladaptive and impact the quality of care provided to individuals living with dementia. This study explores caregiver demographics, the caregiving context, resentment toward the individual with dementia, and caregiver readiness as predictors of dementia management strategies. Participants included 200 family caregivers for a person living with dementia. Descriptive statistics and adjusted linear regressions were run to examine associations between caregiver demographics, the care context (dementia diagnosis, dementia severity rating scale), the Sense of Competence Care Recipient Satisfaction Scale, and the Caregiver Readiness Scale with three outcomes: criticism, active management, and encouragement (Dementia Management Strategies Scale). Caregivers were 58 years old on average, 82% female, 28% non-White, and 38% adult daughters. In models including caregiver demographics and the care context, caregiver readiness consistently related to dementia management as it was associated with less criticism (B=-0.23, p<.01), more active management (B=0.26, p<.01), and more encouragement (B=0.20, p<.01). Greater financial difficulty (B=2.57, p<.05), less dementia severity (B=-0.17, p<.001), and provision of care for an individual with Alzheimer’s disease (relative to early-onset Alzheimer’s disease) (B=-3.01, p<.05) were associated with more encouragement. Further, individuals with more resentment toward the individual with dementia used more criticism (B=0.86, p<.001). Readiness for care appears to be a key modifiable intervention target to improve care management quality provided by family caregivers. Caregiver demographics did not relate to care management once the caregiving context was accounted.

